# Effects of Perceptual-Cognitive Training on Anticipation and Decision-Making Skills in Team Sports: A Systematic Review and Meta-Analysis

**DOI:** 10.3390/bs14100919

**Published:** 2024-10-09

**Authors:** Ruihan Zhu, Man Zheng, Shuang Liu, Jia Guo, Chunmei Cao

**Affiliations:** Division of Sports Science and Physical Education, Tsinghua University, Haidian District, Beijing 100084, China; zrh22@mails.tsinghua.edu.cn (R.Z.); zhengm20@mails.tsinghua.edu.cn (M.Z.); 13910222848@163.com (S.L.); guojia24@mails.tsinghua.edu.cn (J.G.)

**Keywords:** perceptual-cognitive training, on-court transfer effect, optimal training methods, decision-making and anticipation, meta-analysis

## Abstract

Team sports require athletes’ exceptional perceptual-cognitive skills, such as anticipation and decision-making. Perceptual-cognitive training in laboratories aims to enhance these abilities. However, its effectiveness in real-game performance remains controversial, necessitating a systematic review and meta-analysis to determine optimal training methods. Following the PRISMA guidelines, we searched databases (e.g., PubMed, WOS, Scopus, and EBSCO) for relevant studies published before November 2023, assessed study quality, extracted important characteristics, and conducted a meta-analysis using Stata 15.1. This study was registered in PROSPERO (CRD42023494324). A total of 22 quantitative studies involving 45 effect sizes were included. Perceptual-cognitive training positively influenced elite athletes’ anticipation and decision-making. However, its transfer effect on real-game performance improvement (*ES* = 0.65) was inferior to laboratory performance improvement (*ES* = 1.51). Sub-group analyses indicated that the effects of training interventions varied based on stimulus presentation and intervention duration. Based on our findings, we concluded that while perceptual-cognitive training improved on-court performance, its transfer effects were limited. To maximize effectiveness, future interventions should use virtual reality to present training stimuli and incorporate participants’ sport-specific responses to reflect real-game scenarios.

## 1. Introduction

Team sports involve intense rivalry and intricate tactical interactions. In the dynamic and open environment of a game, athletes must continuously monitor and adapt to rapidly changing conditions, such as positional relationships between teammates and opponents, and respond appropriately within a constrained timeframe based on the current situation [1,2]. Elite athletes require outstanding perceptual-cognitive abilities, including advanced anticipation and decision-making skills, to navigate the rapid pace and intensity of team sports [3]. Anticipation denotes the ability to discern the outcome of opponents’ actions prior to their execution, such as predicting the direction of a volleyball spike [4]. Decision-making involves identifying and synthesizing environmental cues using existing knowledge to generate effective actions in dynamic competitive environments [5]. These two skills enable athletes to respond appropriately, resulting in excellent sports performance [6]. Expert–novice studies indicated that elite athletes both outperform novices in real games and demonstrate superior cognitive skills in laboratory-based sports-specific cognitive tasks [7], as evidenced by their higher accuracy and faster response times [8].

Currently, athletes’ tactical knowledge and decision-making skills improve primarily through self-learning experiences during on-court training and competition [9]. However, on-court training has drawbacks: it requires high-intensity activity and is physically demanding. This could lead to unnecessary fatigue and injuries. Additionally, the variable and uncontrollable nature of in-game scenarios makes it challenging to isolate specific decision-making situations [10]. To overcome these limitations and effectively improve athletes’ cognitive skills, numerous studies have examined whether isolated laboratory-based cognitive training can improve athletes’ anticipation and decision-making and whether these improvements can be transferred to actual competition performance [11]. In laboratory settings, decision-making training takes various forms [12]. Among these, the most prevalent and effective method is perceptual-cognitive training, especially video-based training [13], in which videos are used to display stimuli or sports scenarios that necessitate participants’ perceptual reactions [14]. Through video-based training, coaches can control specific anticipation decision-making scenarios to effectively improve athletes’ tactical knowledge and develop their attention toward pertinent perceptual cues [10]. Three-dimensional multiple object tracking (3D-MOT) training is a visual training form aimed at improving visual attention, information processing speed, and working memory [7], which are critical for team sports performance. To be sure, 3D-MOT tasks can effectively replicate real-game scenarios. For example, in team sports such as soccer or basketball, during offensive and defensive play, athletes need to simultaneously monitor and process the positions and movements of multiple opponents and teammates. Moreover, the correlation between 3D-MOT and real-game performance has been strongly demonstrated [7], affirming the efficacy of this training.

Current intervention studies consistently indicate that isolated perceptual-cognitive training can effectively enhance athletes’ cognitive skills in laboratory-based tasks. However, conclusions regarding the effectiveness of transferring these skills to on-court performance (the transfer effect) remain controversial [7]. The ultimate goal of such training is to improve athletic performance effectively in real competitions. Therefore, investigating whether the transfer effect exists and exploring optimal methods to train transfer performance are crucial focuses for sports training and research. Theoretically, scholars generally agree that the representativeness of the training-provided stimulus in real-game competition scenarios (i.e., ecological validity) is crucial for the on-court transfer effect [15]. The Modified Perceptual Training Framework (MPTF), proposed by Hadlow, is a widely recognized classic model based on representative learning design [16]. This framework suggests that the following three ecological factors could influence the transfer effect of perceptual training: the targeted ability of the training must correspond to the perceptual-cognitive functions required in real competitions; the stimuli videos used during training should resemble the scenarios athletes encounter in actual competitions; and the responses required during training should closely align with those required on the court, demanding athletes perform specific actions such as defensive movement to the left or right, passing, shooting, or hitting the ball [17].

Numerous experimental intervention studies have tested the transfer effectiveness of perceptual-cognitive training; however, the findings are controversial, typically because of small sample sizes. Therefore, a systematic review and meta-analysis are necessary to pool data and provide a more robust assessment of training effectiveness [12]. Previous systematic reviews demonstrated significant positive effects of training on laboratory-based anticipation and decision-making skills. However, these reviews had some limitations. First, rather than analyzing on-court transfer performance and laboratory-based scores as separate outcome measures, they only considered laboratory decision-making performance or conflated the two [12]. Second, some reviews only focused on a particular sport [11], resulting in a limited number of studies. Third, some studies lacked a quantitative meta-analysis, offering only qualitative systematic reviews [10,14].

To address these limitations, this review assessed whether improvements in anticipation and decision-making from perceptual-cognitive training in elite athletes could be transferred to on-court sports performance. We conducted a systematic review and meta-analysis, including both randomized controlled trials (RCT) and non-randomized studies (NRS), to quantitatively explore the transfer effect of this type of training. Further, we systematically extracted and categorized the characteristics of each study using a characteristic table and conducted a series of sub-group meta-analyses to investigate how different characteristics, such as ecological factors, influence transfer effects. Through our analyses and discussions, we aimed to identify the optimal intervention form, guide future research, and provide practical recommendations for future perceptual-cognitive training in elite athletes.

## 2. Materials and Methods

In our systematic review and meta-analysis, we followed the guidelines of the 2009 Preferred Reporting Items for Systematic Reviews and Meta-analysis (PRISMA) checklist [18] (Table S1). This systematic review was registered with the International Prospective Register of Systematic Reviews (PROSPERO; CRD42023494324).

### 2.1. Search Strategy

In November 2023, after summarizing keywords from previous reviews [10,11,12] and discussions, we ultimately determined the search strategy used to retrieve relevant studies using various databases, including PubMed, Web of Science, Scopus, SPORTDiscus, and PsychInfo (via EBSCO). Following the population, intervention, comparison, outcomes, and study design (PICOS) approach, the search strategy included participants, interventions, and study types. The search criteria included full-text availability, publication date, and language (Appendix A). The reference lists of the included studies were manually reviewed to identify additional suitable studies.

### 2.2. Selection Criteria

After conducting the literature search, we imported the retrieved studies into reference manager software (Zotero 6.1) and removed duplicate entries. Two authors (Zhu R. and Zheng M.) independently reviewed all the articles. Non-relevant studies were initially excluded by screening titles and abstracts and downloading the full texts of potentially suitable articles [19]. More noncompliant studies were excluded after reading the full text. Upon completing the independent review, the reviewers sought to reach a consensus concerning which studies would be included. In cases of disagreement, a third reviewer (Cao C.) was consulted to make a final decision. Ultimately, all three reviewers agreed on the final selection of studies.

#### 2.2.1. Inclusion Criteria

Inclusion criteria were developed according to PICOS study design principles [20,21]:

1. Participants: Elite team sports athletes with more than three years of team sports experience, not limited to age.
2. Interventions: Participants underwent cognitive-perceptual training (such as MOT or video-based training) programs to develop sports-specific anticipation or decision-making skills.
3. Comparators: Control groups (passive control, no extra training except regular on-field training) or placebo groups (active control, watching videos of the same duration as training groups, but not performing meaningful training).
4. Outcomes: At least one of the following indicators—laboratory task-specific response accuracy (RA), response time (RT), or on-court transfer RA/performance RT.
5. Study design: RCT with pre-tests and post-tests or NRS with pre-tests and post-tests.

#### 2.2.2. Exclusion Criteria

We excluded studies based on the following characteristics:

1. Participants were individual sportspersons or beginners who had not participated in professional training or official events;
2. The intervention group received on-field training (such as a mini-tournament or small-sided game) rather than cognitive-perceptual training;
3. The study lacked a control/placebo group;
4. The full text was not available, the study data (pre-test and post-test mean [M] and standard deviation [SD]) could not be extracted and calculated, and the data remained unavailable after contacting the corresponding authors;
5. Qualitative research, reviews, non-intervention studies, dissertations, and conference papers.

### 2.3. Data Extraction

Two researchers (Zhu R. and Zheng M.) independently extracted the attributes and data of the included studies and recorded them in a pre-established standardized table following the PICOS methodology. Where necessary, a third senior researcher (Cao C.) was consulted to validate the decisions taken.

### 2.4. Assessment of Methodological Quality and Heterogeneity

**Figure 1:**
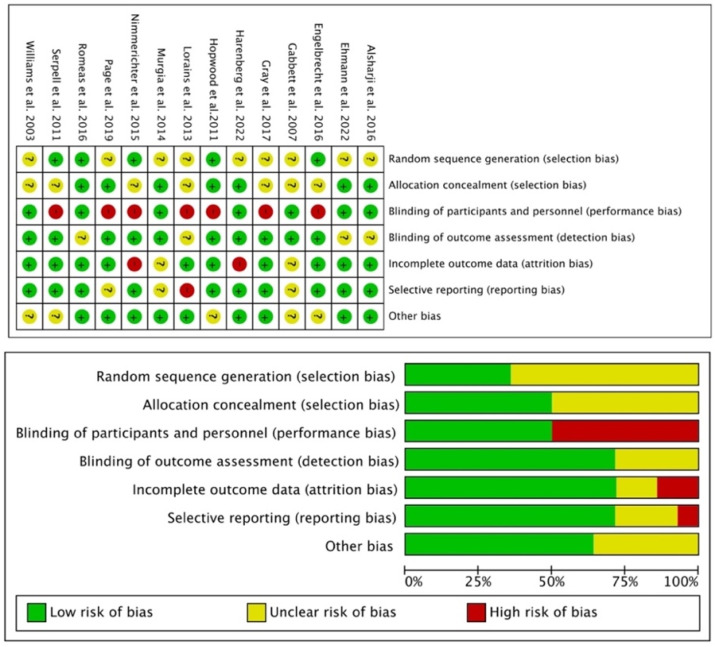
Cochrane Risk of Bias (RoB) graphs of the RCT studies. Notes: “+”, low risk of biase, “?”, unclear risk of bias, “-“, high risk of bias [7,24,25,26,27,28,29,30,31,32,33,34,35,36].

The researchers independently evaluated the methodological rigor and potential bias of all the studies. We employed the Cochrane Risk of Bias (RoB) tool [22] in Revman5.3 software for the 14 included RCT studies. The risk of bias was assessed for seven criteria, and each criterion was rated as low, unclear, or high risk, culminating in the creation of RoB graphs (Figure 1). The results indicated that owing to insufficient rigor in the methodology, there was publication bias in the included studies. Regarding selection bias, only two studies provided detailed descriptions of how random sequence generation and allocation concealment were conducted, with most other studies having at least one aspect with an unclear or high risk of bias. Second, seven studies did not consider double blinding, potentially resulting in a high risk of detection bias. Four studies did not explain the reasons for participants’ high dropout rates, causing risks of attrition bias. Three studies selectively reported data for some outcomes—for example, reporting only F- or *p*-values—resulting in a high or unclear risk of reporting bias.

For NRS, the Risk of Bias in Non-randomized Studies of Interventions (ROBINS-I) tool [23] was used to assess the potential bias by consulting prior research [12]. This assessment tool comprises three dimensions: pre-, at-, and post-intervention bias. The overall risk of bias for each study was determined and categorized as low, moderate, or critical, as summarized in the ROBINS-I tables (Table 1). Overall, three studies were rated as critical risk, four as moderate risk, and one as low risk.

Additionally, we used funnel plots to depict heterogeneity intuitively among the included studies (Appendix B), which showed that some dots were outside the dashed line, indicating heterogeneity among the studies, especially for outcomes like task-specific RA and on-court transfer RA. The sensitivity of the analysis was assessed using trim plots (Appendix C), which showed that none of the 95% confidence intervals (CIs) deviated from the original intervals, indicating that our meta-analysis was stable.

**Table 1:** The Risk of Bias in Non-randomized Studies of Interventions (ROBINS-I) table of the included NRS.

Author and Year	Pre-Intervention		At Intervention	Post-Intervention				Overall
	Confounding	Selection of Participants	Interventions Classification	Deviations from Intended Interventions	Missing Data	Measurement of Outcomes	Selection of Reported Results	
Gabbett et al., 2008 [37]	Low	Low	Low	Low	Low	Low	Moderate	Low
Gorman et al., 2009 [17]	Moderate	Low	Low	Moderate	Moderate	Low	Moderate	Critical
Janvier et al., 2010 [38]	Low	Moderate	Moderate	Low	Moderate	Low	Low	Critical
Smeeton et al., 2013 [39]	Low	Low	Moderate	Low	Low	Moderate	Low	Moderate
Hohmann et al., 2016 [40]	Moderate	Low	Low	Low	Low	Low	Moderate	Moderate
Holding et al., 2017 [41]	Low	Low	Low	Low	Low	Low	Low	Low
Panchuk et al., 2018 [42]	Moderate	Critical	Moderate	Low	Low	Moderate	Low	Critical
Sáez-Gallego et al., 2018 [43]	Low	Low	Moderate	Moderate	Low	Low	Low	Moderate

Notes: Risks are categorized as low, moderate, and critical, in ascending order of severity. Finally, “overall” pertains to a comprehensive summary of the quality assessment and the overall risk of bias for the entire article.

### 2.5. Statistical Analysis

#### 2.5.1. Summary Measures and Effect Size (ES) Calculation

To quantify the effects of perceptual-cognitive training on anticipation and decision-making skills, we conducted a meta-analysis using Stata SE 15.1 software, which primarily focused on four main outcomes: athletes’ decision RA and RT in laboratory-based tasks, as well as the on-court transfer of RA and RT to the real sports competition. M and SD values of the pre- and post-intervention tests were calculated. Corresponding values reported directly in the text were preferred. If unavailable, we estimated values from other reported data or those obtained by contacting the corresponding author; articles or data that could not be obtained were excluded. Because pre-test values may differ among different groups in some studies, rather than solely relying on post-test values, we calculated pre- and post-test differences (mean difference, MD) and their standard deviation (SD_MD_) by adhering to the formulas recommended by the Cochrane guidelines [22] for each group and included this in the meta-analysis.

Stata SE 15.1 software was used to conduct the meta-analysis because of the large variation in the mean values of the outcomes and the small sample size of the included studies. We adjusted for bias when calculating effect sizes using Hedges’ g, expressed as effects size (ES) with 95% CIs [44,45]. The ESs were categorized as small (0.2), medium (0.5), or large (0.8) based on their absolute values [46]. The heterogeneity was tested, with I-squared (*I*^2^) < 50% indicating low heterogeneity and *I*^2^ > 50% indicating high heterogeneity [47,48]. Since the research papers included in this study involved various sports, there was inherent heterogeneity among the studies. Therefore, we uniformly used a random-effects model for analysis.

#### 2.5.2. Sub-Group Analysis

We used Stata SE 15.1 software for sub-group effects analysis for on-court transfer RA, which was undertaken to investigate whether the impact of the intervention on on-court transfer decision-making abilities was attributable to varying training characteristics such as age, stimulus presentation equipment type (computer screen, life-size projector screen, or 3D/virtual reality [VR] device), required response (verbal response or sport-specific action response), duration of the entire intervention (≤4 weeks or >4 weeks), or frequency (<3 sessions/week or ≥3 sessions/week).

## 3. Results

### 3.1. Study Identification and Selection

**Figure 2:**
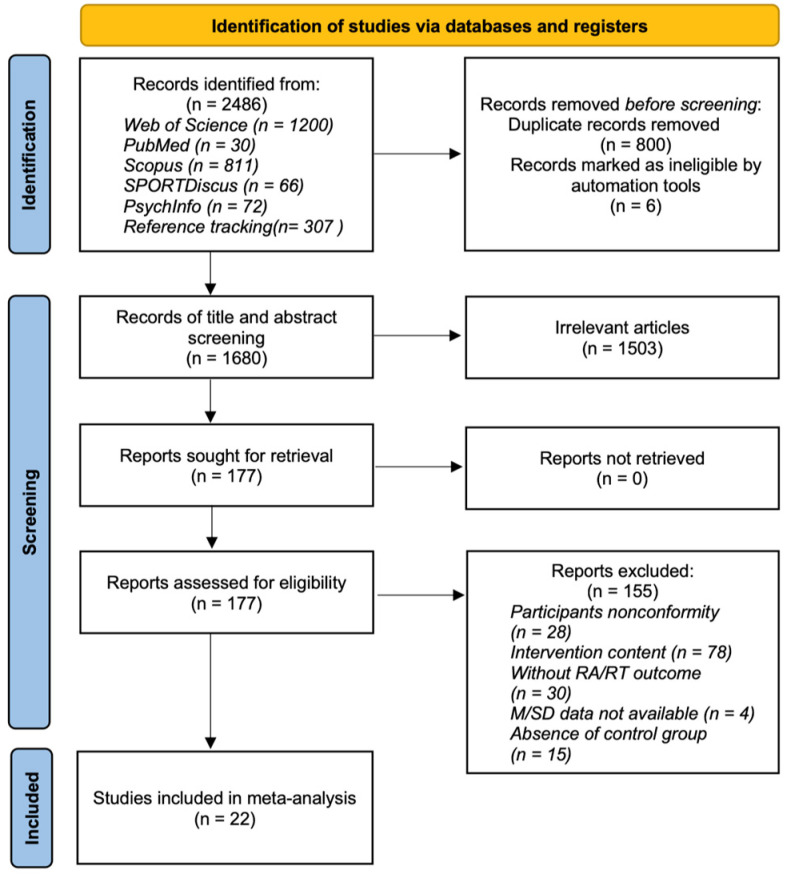
The PRISMA flow chart. Notes: RA, response accuracy; RT, response time; M, mean; standard deviation, SD.

After selection, 22 eligible studies were included in this systematic review and meta-analysis (Figure 2).

### 3.2. Characteristics of the Included Studies

We extracted data from 22 of the included studies using the PICOS methodology. In Table 2, we first extracted participants’ main characteristics (country, team sport type, expertise level, age, and sample size of each group) and study design (RCT or NRS) for each study (Table 2). Additionally, we extracted in detail the outcome indicators included in the studies and the methods used to measure on-court transfer skills (Table 2). Table 3 shows the main characteristics of the intervention (type, frequency, and duration) and comparison (placebo or control group). For more detailed characteristics, refer to Table S2.

#### 3.2.1. Participants

Relevant studies have examined various sports teams (Table 2). Among these, soccer garnered the most attention, with seven included studies. This is likely because of the popularity of soccer and its extensive research resources. Additionally, soccer involves complex tactics requiring superior perceptual-cognitive skills [7,33]. Rugby ranked second in the number of studies included, with four articles. According to expertise levels, participants in the included studies met the experience standards of elite athletes: one study focused on international-level athletes, seven studies focused on national-level athletes, five focused on professional-level athletes, six focused on university- or high-school-level athletes, and three focused on club-level athletes. Regarding sex and age, four studies focused solely on female athletes, ten focused solely on male athletes, and eight focused on both or did not report specific sex. Thirteen studies targeted adults (>18 years), whereas nine focused on young athletes (14–18 years).

#### 3.2.2. Intervention vs. Comparison

For the intervention forms (Table 3), the types of stimulus presentation can be divided into three main categories: seven computer videos, eight projectors for life-size videos, and seven VR devices for immersive stimuli. Regarding the response type, 11 studies required verbal responses or keyboard clicking, and 11 required specific action responses, which more closely simulated real scenarios. Additionally, owing to the absence of standardized scientific guidelines for cognitive-perceptual training [28], there were variations in duration and frequency across different interventions. The included studies indicated that the total intervention periods ranged from 1 to 8 weeks, with frequencies varying from once a week to seven times a week, and each session lasted between 5 and 45 min. For comparison, some studies established a placebo group by having participants watch ordinary competition videos [29,31] or engage in non-sports cognitive tasks [17,25], whereas others only set up a negative control group with no intervention.

#### 3.2.3. On-Court Transfer Measurement

Two primary methods were employed (Table 2) to measure on-court transfer abilities. The first method directly transformed video stimuli into actual situations and calculated their success rates in a real court [38]. The second method involved placing participants in small-sided games [33] in which coaches used coding instruments to assess player decision-making skills quantitatively.

### 3.3. Total ES: Intervention vs. Control/Placebo

**Figure 3:**
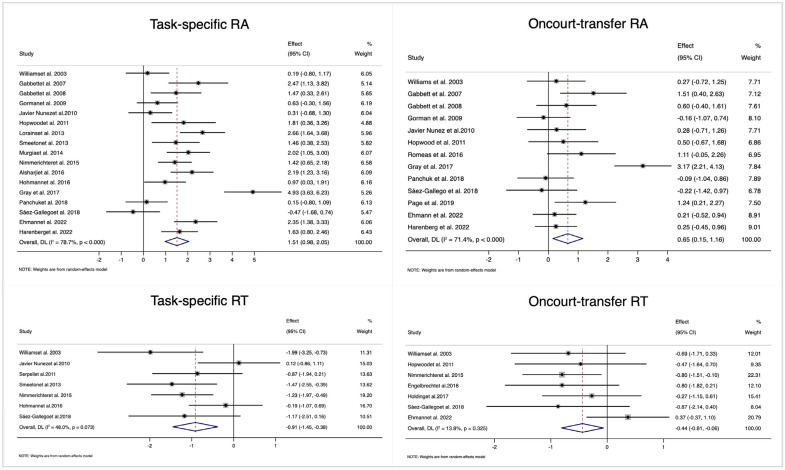
Forest plots depicting the total effect size (ES) of interventions on four outcomes. Notes: RA, response accuracy; RT, response time; CI, confidence interval [7,17,24,25,26,29,30,31,32,33,34,35,36,37,38,39,40,41,42,43].

Among the 22 studies included in this systematic review, 17 provided task-specific RA (pooled *n* = 381), 13 provided on-court transfer RA (pooled *n* = 247), 8 provided task-specific RT (pooled *n* = 130), and 7 provided on-court transfer RT (pooled *n* = 139). Figure 3 illustrates the effects of training on these four outcomes. There was a large effect (ES = 1.51, 95% CI = [0.98, 2.05], *I*^2^ = 78.7%) for task-specific RA (upper left) but only a medium-sized effect (ES = 0.65, 95% CI = [0.15, 1.16], *I*^2^ = 71.4%) for on-court transfer RA (upper right). As a shorter RT may indicate better cognitive skills, the ESs were usually negative. Therefore, we considered the absolute value of the ES for RT. For task-specific RT (lower left), the ES was large (ES = −0.91, 95% CI = [−1.45, −0.38], *I*^2^ = 48.0%), favoring the intervention groups, and there was only a close to medium ES (ES = −0.44, 95% CI = [−0.81, −0.06], *I*^2^ = 13.8%) for on-court transfer RT (lower right).

### 3.4. Sub-Group Analysis of the Transfer Effect

#### 3.4.1. Age

For transfer RA, there were medium-sized effects for both adult (ES = 0.63, 95% CI = [0.29, 0.98], *I*^2^ = 0.0%) and adolescent (ES = 0.58, 95% CI = [−0.65, 1.82], *I*^2^ = 88.7%) groups, with no significant heterogeneity between them (*p* = 0.939).

#### 3.4.2. Ecological Factors Affecting the Intervention

**Figure 4:**
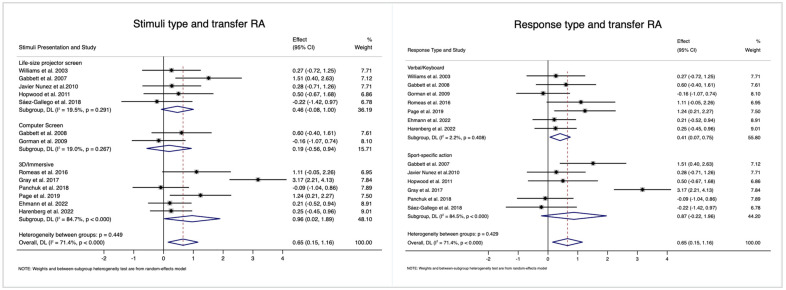
Sub-group analyses testing the different transfer effects of intervention among different stimuli types or response types. Notes: RA, response accuracy; RT, response time; CI, confidence interval [7,17,24,25,26,33,34,35,36,37,38,42,43].

The effects of different ecological training factors on RA transfer are shown in Figure 4. The ESs on transfer RA differed among the stimulus types, but the difference was not significant (*p* = 0.449). For computer videos, there was only a small-sized effect (ES = 0.19, 95% CI = [−0.56, 0.94], *I*^2^ = 19.0%). For life-size videos, there was a close to medium-sized effect (ES = 0.46, 95% CI = [−0.08, 1.00], *I*^2^ = 19.5%). For 3D/VR training, the ES was significantly larger (ES = 0.96, 95% CI = [0.02, 1.89], *I*^2^ = 84.7%).

It is universally acknowledged that participants’ responses to stimuli may also influence the transfer effect. For transfer RA, the ES was small (ES = 0.41, 95% CI = [0.07, 0.75], *I*^2^ = 2.2%) when participants’ verbal responses were required; however, there was a large effect (ES = 0.87, 95% CI = [−0.22, 1.96], *I*^2^ = 84.5%) when sports-specific action responses were required. However, no significant heterogeneity was observed among the different response types (*p* = 0.429).

#### 3.4.3. Duration and Frequency of Training Interventions

**Figure 5:**
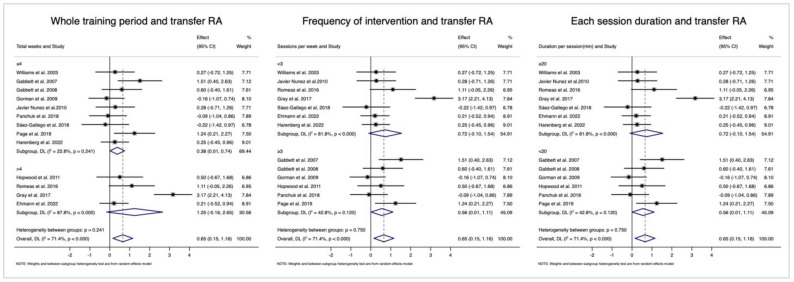
Sub-group analyses testing the different effects among different durations of whole training periods (**left side**), frequency of intervention (**middle section**), and each session duration (**right side**). Notes: RA, response accuracy; RT, response time; CI, confidence interval. [7,17,24,25,26,33,34,35,36,37,38,42,43].

Figure 5 (left side) indicates that studies with longer training periods had a larger effect on transfer RA (≤4 weeks: ES = 0.38, 95% CI = [0.01, 0.74], *I*^2^ = 22.8%; >4 weeks: ES = 1.25, 95% CI = [−0.16, 2.65], *I*^2^ = 87.8%) but the difference was not significant (*p* = 0.241). For each session (Figure 5, middle section), the effect was medium for both shorter (<20 min: ES = 0.56, 95% CI = [0.01, 1.11], *I*^2^ = 42.8%) and longer (>20 min: ES = 0.72, 95% CI = [−0.10, 1.54], *I*^2^ = 81.8%) durations. For the training frequencies (Figure 5, right side), the ESs were similar for both groups (<3 sessions/week: ES = 0.72, 95% CI = [−0.10, 1.54], *I*^2^ = 81.8%, ≥3: ES = 0.56, 95% CI = [0.01, 1.11], *I*^2^ = 42.8%).

## 4. Discussion

This study systematically reviewed the scientific literature on the effects of perceptual-cognitive training on elite athletes’ anticipation and decision-making skills. Overall, the intervention groups showed significant improvements in both RA and RT compared with the control groups, both in laboratory-based tasks and on-court performance, which aligns with previous findings [10,12]. This type of training targets sports-specific tactical situations [50] and guides athletes to accurately capture the most critical information in the environment at optimal moments, thereby enhancing their ability to respond correctly in shorter times [51,52]. However, while training had a large effect on laboratory-based outcomes, it only showed medium-sized effects on transfer outcomes, indicating that while perceptual-cognitive training can enhance real-game decision-making performance to some extent, its transfer effects may be limited.

### 4.1. Ecological Factors: Key Influences on Training Transfer Effectiveness

The MPTF [16,53] posits that the representativeness and ecological validity of perceptual training are crucial for transfer effectiveness [7]. Therefore, we conducted sub-group analyses to explore how different ecological factors in intervention training influence the transfer effect. Our findings support the MPTF.

First, the included studies aimed to improve the decision-making skills required in competition, in line with the first aim of the MPTF. Second, regarding the stimulus presentation type, the transfer effect increased when stimulus fidelity was higher. In early perceptual training, computer screens were commonly used as stimulus presentation devices [17,37]; however, the results indicated that computer video training produced the smallest transfer effects. This could be owing to the small size of the screens, which failed to adequately simulate real-game environments [28]. Subsequent studies have used projectors to display life-size 2D videos, yielding a medium-sized transfer effect [26,31]. In the 2D life-size video training, the most effective type of training stimuli was the “first-person perspective” video, created by equipping a player with a head-mounted sports camera to capture their view of the court [10,35]. This approach significantly outperformed third-person perspective videos shot from fixed positions on the court [4], since first-person videos better simulate the actual visual situation on the court, helping participants develop better self-perception [54]. With technological improvements, 3D and VR devices have been introduced in perceptual training [33,42] to create simulated real-game settings, offering an immersive, multisensory environment that allows athletes to engage interactively [55,56]. Our sub-group analysis results indicated the highest transfer effect for studies using 3D and VR devices. For instance, some studies had participants wear head-mounted VR devices and watch and respond to videos of offensive decision-making scenarios taken from the first-person perspective of basketball players [35]. Other studies involved participants watching immersive 3D circular screen videos or engaging in 3D-MOT tasks [7,36]. Using VR devices to display first-person perspectives provided the most realistic visual simulation of actual competition [10]. Finally, our findings demonstrated that training incorporating sports-specific movement responses is more effective in enhancing transfer performance compared to training that only requires verbal responses. This is because sports-specific action responses during training can enhance perception-action coupling [57], thereby enabling participants to apply the skills acquired in training more effectively in real competitive scenarios, resulting in quicker and more accurate responses [58].

Regarding training duration and frequency, when the total training period exceeded 4 weeks, it was more effective. Conversely, acute intervention was less effective. This accords with previous findings [11]. Although some previous studies indicate that twice-weekly training interventions are more effective, our results did not show any differences at different frequencies. Interestingly, we observed a correlation between frequency and single-session duration, with studies tending to have longer single-session training durations (≥20 min) when the frequency of the intervention was low (<3 sessions/week), which we speculate was to ensure that the total training duration per week was at a similarly reasonable level. Additionally, interventions with a single training session duration (5–10 min) were too short to be effective [41,42].

### 4.2. Comparative Characteristic Summary of the Details of Other Training Procedures

Temporal occlusion is frequently employed in video training, wherein videos are paused at a certain frame before the athlete’s action, requiring immediate participant responses, rather than playing through to completion [53]. This approach helps develop athletes’ perceptual-cognitive patterns as they can efficiently capture early critical information, significantly improving both the accuracy and timeliness of their responses [59,60]. Offensive decision-making requires players to judge and execute tactics in a complex, dynamic environment [61]. Typically, only a single temporal occlusion point set just before a decision is required for offensive decision-making training to display complete tactical information that aids accurate decision-making [28,37]. However, defensive players must focus on their opponent’s physical kinesiology-related information to anticipate their possible intentions [6,8], and experts can anticipate opponents’ actions earlier than novices [4,62]. In a training study [25] on softball batting direction prediction, three temporally occluded videos were set up, including pre-contact, during-contact, and post-contact, to train participants to progressively capture essential information earlier. Alsharji [31] employed both spatial and temporal occlusion techniques to enhance training in anticipation skills among handball athletes. The performance of the intervention groups in these two studies improved significantly, indicating that employing multiple types of occlusion videos may further enhance anticipation skills.

Some studies have used an explicit instructional approach to help participants pay attention to key information in video stimuli by providing instructions during training [24]. Instructions can take various forms, such as using arrows to point to relevant areas during video play [24,27,41] or directing attention through verbal instruction before video play [17,37]. In contrast, in the implicit training approach, participants received no instructions and relied on their perceptions to identify relevant information [63]. Some studies compared explicit and implicit interventions [17,41] and found no significant difference between the two forms in enhancing the ability to recognize and process task information [52,64].

Most interventions typically used fixed-speed video stimuli; however, there were some exceptions. Some studies used 1.5× more real-time videos [28] to train defensive anticipation skills. They found that accelerated video training was more effective for overall ability improvement and long-term retention (after 10 weeks) than regular-speed video training. A speed of 1.5 may be optimal for video training, as it increases the urgency of tasks, forcing athletes to respond more automatically and with less time for information processing, thereby enhancing their on-court urgent response [65]. Further, 3D-MOT [33,36] and some 3D video training [34] used adaptive training techniques, adjusting the stimulus speed through a staircase procedure based on participants’ RA during training. This training method better simulates different conditions that may be encountered in a real game, prompting participants to explore the perceptual-motor space more deeply [66,67].

Our results did not show any differences in the transfer effects between adults and adolescents. A critical stage of decision-making skill development occurs during early adolescence [12,68] and adolescents will reach a stable and practically effective level at 15 years of age [69]. The young participants included in that study were elite athletes with significant training experience. Additionally, they were primarily older than 15 years, at which time their tactical decision-making skills may be developing at a rate close to that of adults. This could explain the non-significant differences between them and adults in terms of training effects [12].

### 4.3. Limitations of Existing Studies and This Review

Most of the included studies involved training using computer videos that did not involve an on-court transfer test. Additionally, only a limited number of studies could be included in the meta-analysis of on-court transfer outcomes [69]. Accordingly, our discussion on the training transfer effect of computer videos was relatively limited [16], which may have led to high meta-analysis estimates of the total ESs. Only half of the reviewed studies included a placebo group to control for the intervention expectancy effect, thus ensuring objectivity [69,70,71]. A retention test is crucial because it can determine whether there is a potential sustained effect of the training [10,72]; however, only seven of the included studies established retention tests, and the time points of the retention tests were not unified. The reporting of outcome data (e.g., M or SD) in some studies was not standardized, which could cause inconvenience or estimation bias in future quantitative meta-analyses. Overall, there has been a lack of standardization and a high risk of bias in the methodological design used in current intervention studies.

This systematic review had some limitations. It only included the literature written in English; therefore, relevant studies written in other languages were not analyzed. Additionally, to extract more consistent outcome metrics (in terms of RA/RT), our review focused mainly on perceptual-cognitive training and ignored training related to tactical reflection and understanding [12], such as self-questioning [73] and imagery training [74]. Finally, our meta-analysis results need to be interpreted with caution because of heterogeneity in study design and methodological quality among the included studies, which may explain why we observed trends in sub-group analyses but non-significant *p*-values.

### 4.4. Future Research and Practical Training Application

Based on the results, we offer the following suggestions for future practical training applications. Perceptual-cognitive training should focus on the fidelity of training stimuli. VR devices should be used to play videos from a first-person perspective and require athletes to respond to sports movements. This type of training was the most effective. We suggest that the total training period should be more than four weeks, with approximately two to four sessions per week, and each session should last between 10 and 25 min. This type of intervention should be performed regularly on athletes to prevent possible recessionary effects.

## Figures and Tables

**Table 2:** The main characteristics of the participants (P), outcomes (O), and study design (S).

Author and Year	Country	Study Design	Team Sport	Expertise Level (Training Yrs)	Gender	Age (M ± SD)	N (Sample Sizes)		Outcomes Extracted	How to Test and Measure the On-Court Transfer Skills?	Retention Test or Not?
							Intervention	Control			
Williams et al., 2003 [24]	UK	RCT	Field hockey	University level (8.5 ± 2.2)	F	21.6 ± 2.2	VT: 8	P *: 8 C: 8	Task RA and RT Transfer RA and RT	The response accuracy and time (RA and RT) for defense of real penalty kicks	No
Gabbett et al., 2007 [25]	Australia	RCT	Softball	National/State level	F	19 ± 1	VT: 9	P *: 8 C: 8	Task RA Transfer RA	The RA and RT for anticipating and catching real pitcher throws	Retention (4 weeks)
Gabbett et al., 2008 [37]	Australia	NRS	Soccer	Professional/ National level	F	18.3 ± 2.8	VT: 8	C: 8	Task RA Transfer RA	Assessed decision-making using modified coding criteria by scientists blinded to the training protocol in the small-sided games (SSG)	No
Gorman et al., 2009 [17]	Australia	NRS	Basketball	Professional level (7.7 ± 3.4)	F/M	17.8 ± 2.1	EVT *: 10 IVT: 10	P *: 9 C: 10	Task RA Transfer RA	Assessed by the investigator using the coding instrument in the actual competition	Retention (2 weeks)
Javier Nunez et al., 2010 [38]	Spain	NRS	Soccer	Professional level (>10 yrs)	M	23.2 ± 2.2	VT *: 8 DT:8	P *: 8 C: 8	Task RA and RT Transfer RA	The success-goal rate (RA) of real penalty kicks	Retention (1 day) Retention (1 week)
Hopwood et al., 2011 [26]	Australia	RCT	Cricket	International level	M	21.3 ± 2.6	VT: 7	C: 5	Task RA Transfer RA and RT	The RA and RT for real batting by facing a bowling machine	No
Serpell et al., 2011 [27]	Australia	RCT	Rugby	National level	F/M	>18	VT: 8	C: 7	Task RT	No transfer test, only a laboratory test	No
Lorains et al., 2013 [28]	Australia	RCT	Rugby	>3.2 yrs at AFL Professional level	F/M	22.3	FVT *: 16 NVT: 15	C: 14	Task RA	Assessed decision-making using coding instruments in real games (M ± SD data cannot be extracted)	Retention (2 weeks) Retention (10 weeks)
Smeeton et al., 2013 [39]	UK	NRS	Cricket	National level	M	14.9 ± 0.75	IT: 8 VT *: 7	C: 10	Task RA and RT	No transfer test, only a laboratory test	No
Murgia et al., 2014 [29]	Italy	RCT	Soccer	Professional/Semi-professional level (9.3 ± 2.6)	M	16.0 ± 1.9	VT: 13	P *: 13 C: 12	Task RA	No transfer test, only a laboratory test	No
Nimmerichter et al., 2015 [30]	Austria	RCT	Soccer	University level (3–5 yrs)	M	14.4 ± 0.1	VT: 18	C: 16	Task RA and RT Transfer RT	The RT of Reactive-agility Sprint Test in the soccer field (react ASAP by sprinting either left or right)	No
Alsharji et al., 2016 [31]	USA	RCT	Handball	National level (7.33 ± 1.24)	F/M	16.8 ± 0.98	VT: 14	P *: 14 C: 14	Task RA	No transfer test, only a laboratory test	No
Engelbrecht et al., 2016 [32]	South Africa	RCT	Rugby	Recreational/Club level	M	19–23	VT *: 10 FT: 9	C: 7	Transfer RT	The RT of Reactive-agility Sprint Test in the rugby field	Retention (6 weeks)
Romeas et al., 2016 [33]	Canada	RCT	Soccer	University level (12.32 ± 1.01)	M	21.67 ± 0.46	3DMT: 9	P *: 7 C: 7	Transfer RA	Assessed soccer decision-making using modified coding criteria by the researcher in the SSG	No
Hohmann et al., 2016 [40]	Germany	NRS	Handball	National level	M	14.89 ± 0.75	3DVT: 10	P *: 10 C: 10	Task RA and RT	No transfer test, only a laboratory test	Retention (4 weeks)
Holding et al., 2017 [41]	Australia	NRS	Rugby	Professional level	M	14.6 ± 1.09	EVT *: 10 IVT: 10	C: 10	Transfer RT	The RT of Reactive-agility Sprint Test in the rugby field	No
Gray et al., 2017 [34]	USA	RCT	Baseball	High school competitive level (8.5 ± 1.1)	M	17–18	3DAVT *: 20 3DBT: 20 RBT: 20	C: 20	Task RA Transfer RA	% of swings at pitches inside the strike zone (Z-Swing %) in the on-field batting test	Retention (1 month)
Panchuk et al., 2018 [42]	Australia	NRS	Basketball	National level	F/M	17.0 ± 0.6	3DVT: 11	C: 7	Task RA Transfer RA	Assessed basketball skills using SportsCode Elite (Hudl) by the researcher in the 4v4 SSG	No
Sáez-Gallego et al., 2018 [43]	Spain	NRS	Volleyball	Recreational/ Club level (5.88 ± 2.19)	F	17.13 ± 0.89	VT: 6 MT: 5	C: 5	Task RA and RT Transfer RA and RT	The RA and RT for real volleyball jump blocking	No
Page et al., 2019 [35]	Canada	RCT	Basketball	University level (7.0 ± 1.7)	F/M	19.4 ± 3.7	3DVT: 9	P *: 9 C: 9	Transfer RA	Calculated on-court decision scores by coaches in the 5v5 SSG for harmonized settings	No
Ehmann et al., 2022 [7]	Germany	RCT	Soccer	Recreational/ Club level (6.9 ± 2.3)	F/M	12.3 ± 0.7	3DMT: 14	P *: 15 C: 9	Task RA and RT Transfer RA	Assessed decision-making using a coding instrument [49] in the 4 vs. 3+ SSG.	No
Harenberg et al., 2022 [36]	Canada	RCT	Soccer	University level (13.97 ± 2.04)	F/M	19.13 ± 0.92	3DMT: 16	P: 15	Task RA Transfer RA	The RA and RT of soccer ball passing on the real court	No

Notes: (1) RCT: randomized controlled trial; NRS: non-randomized controlled studies; F: female; M: male; F/M: female and male; (2) VT: video-based cognitive-perceptual training; EVT: explicit video-based cognitive-perceptual training; IVT: implicit video-based cognitive-perceptual training; DT: discovery training; FVT: fast-speed video training; NVT: normal-speed video training; IT: imagery training; FT: field training; 3DMT: 3D-MOT (multiple object tracking) training; 3DVT: 3D (virtual environment) video training; 3DAVT: 3D adaptive training in a batting 3D virtual environment (VE); 3DBT: batting practice in the VE without adaptive training; RBT: extra on-field sessions of real batting practice; (3) P: placebo group; C: control group, marked with an asterisk (*) means that the outcome data from this group were used in the meta-analysis; (4) task RA: in laboratory task-specific response accuracy; task RT: in laboratory task-specific response time; transfer RA: on-court transfer response accuracy/score; transfer RT: on-court transfer response time; SSG: small-sided games.

**Table 3:** The main characteristics of the interventions (I) and comparison groups (C).

Author and Year	Type of Training Intervention		Type of Comparison (Placebo/Control)	Duration and Frequency		
	Stimuli	Response		Total Weeks	Sessions/Week	Minutes/Session
Williams et al., 2003 [24]	Life-size Video	Verbal/Keyboard	P *: Instructional video C: No training	1	1	45
Gabbett et al., 2007 [25]	Life-size Video	Specific action	P *: Left/Right arrows C: No extra training	4	3	10
Gabbett et al., 2008 [37]	Computer video	Verbal/Keyboard	C: No extra training	4	3	15
Gorman et al., 2009 [17]	Computer video	Verbal/Keyboard	P *: Non-sport training C: No extra training	4	3	10
Javier Nunez et al., 2010 [38]	Life-size Video	Specific action	P *: Regular sports video C: No extra training	1	1	20
Hopwood et al., 2011 [26]	Life-size Video	Specific action	C: No extra training	6	3	10
Serpell et al., 2011 [27]	Life-size Video	Specific action	C: Common warm-up	3	2	15
Lorains et al., 2013 [28]	Computer video	Verbal/Keyboard	C: No extra training	5	1	15
Smeeton et al., 2013 [39]	Computer video	Verbal/Keyboard	C: No extra training	4	1	25
Murgia et al., 2014 [29]	Computer video	Verbal/Keyboard	P *: Real game TV video C: No extra training	8	1	15
Nimmerichter et al., 2015 [30]	Computer video	Verbal/Keyboard	C: Regular training without extra session	6	2	6
Alsharji et al., 2016 [31]	Life-size Video	Specific action	P *: Real game video C: No extra training	1	7	25
Engelbrecht et al., 2016 [32]	Life-size Video	Specific action	C: Regular training without extra session	6	2	10
Romeas et al., 2016 [33]	3D-MOT	Verbal/Keyboard	P *: Real game video C: No extra training	5	2	24
Hohmann et al., 2016 [40]	3D/VR immersive video	Verbal/Keyboard	P *: Tactical board picture C: No extra training	6	1	30
Holding et al., 2017 [41]	Life-size Video	Specific action	P: Real game video	1	1	8
Gray et al., 2017 [34]	3D/VR immersive video	Specific action	C: Regular training without extra session	6	2	45
Panchuk et al., 2018 [42]	3D/VR immersive video	Specific action	C: Regular training without extra session	3	4	5
Sáez-Gallego et al., 2018 [43]	Computer video	Specific action	C: No extra training	4	2	20
Page et al., 2019 [35]	3D/VR immersive video	Verbal/Keyboard	P *: Computer soccer video C: No extra training	1	4	15
Ehmann et al., 2022 [7]	3D-MOT	Verbal/Keyboard	P *: Real game video C: No extra training	5	2	20
Harenberg et al., 2022 [36]	3D-MOT	Specific action	P: Real game video	4	2.5	25

Notes: P: placebo group; C: control group. Marked with an asterisk (*) means that the outcome data from this group were used in the meta-analysis.

**Figure A1:**
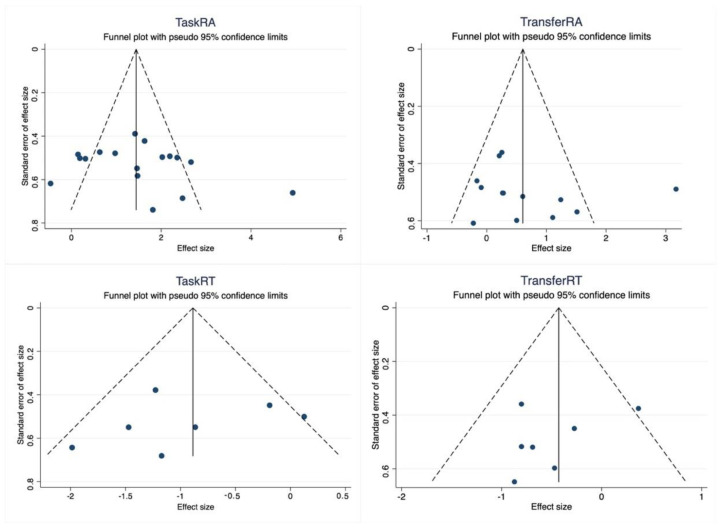
The Funnel Plots for Task RA and RT as well as Transfer RA and RT.

**Figure A2:**
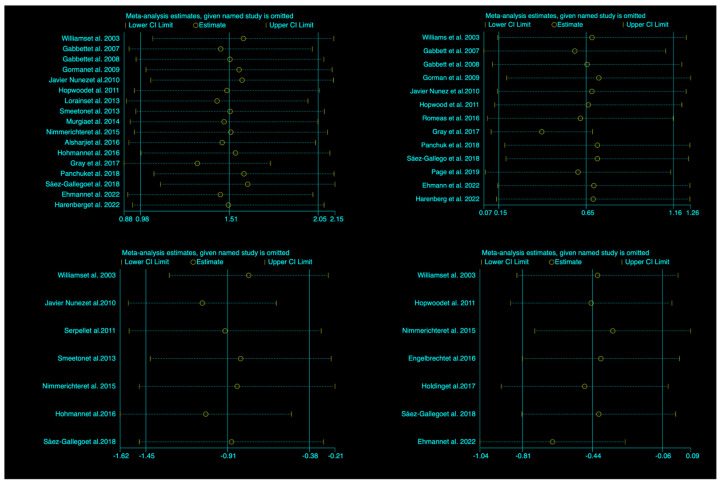
Sensitivity Analysis of Meta-Analysis [7,17,24,25,26,27,28,29,30,31,32,33,34,35,36,37,38,39,40,41,42,43].

## Data Availability

All data in this study are available from the primary research or upon reasonable request from the corresponding author.

## Supplementary Materials

The following supporting information can be downloaded at: https://www.mdpi.com/article/10.3390/bs14100919/s1, Table S1: the PRISMA Checklist of this review; Table S2: detailed characteristics table of included studies. Reference [75] is cited in the Supplementary Materials.

## Appendix A

**Table A1:** Search Strategies.

Database	Search Strategy (Search Date 26 November 2023) Publication Date Must Be before 26 November 2023	Total
PubMed	((“decision training” [Title/Abstract] OR “decision making training” [Title/Abstract] OR “anticipation training” [Title/Abstract] OR “video-based training” [Title/Abstract] OR “VBT” [Title/Abstract] OR “video training” [Title/Abstract] OR “perceptual training” [Title/Abstract] OR “video feedback” [Title/Abstract] OR “visual training” [Title/Abstract] OR “vision training” [Title/Abstract] OR “anticipation” [Title/Abstract] OR “decision making” [Title/Abstract] OR “perceptual cognitive training” [Title/Abstract]) AND (“intervention” [Title/Abstract] OR “experimental” [Title/Abstract] OR “quasi-experimental” [Title/Abstract] OR “experimental group” [Title/Abstract] OR “control group” [Title/Abstract] OR “training” [Title/Abstract])) AND (“team sport” [Title/Abstract] OR football [Title/Abstract] OR soccer [Title/Abstract] OR futsal [Title/Abstract] OR handball [Title/Abstract] OR volleyball [Title/Abstract] OR basketball [Title/Abstract] OR hockey [Title/Abstract] OR rugby [Title/Abstract] OR cricket [Title/Abstract] OR “water polo” [Title/Abstract] OR lacrosse [Title/Abstract] OR softball [Title/Abstract] OR korall [Title/Abstract] OR “American football” [Title/Abstract]). Filters applied: Full text, Clinical Study, Clinical Trial, Observational Study, Randomized Controlled Trial, English.	30
Web of Science	TS=((“decision training” OR “decision making training” OR “anticipation training” OR “video-based training” OR “VBT” OR “video training” OR “perceptual training” OR “video feedback” OR “visual training” OR “vision training” OR “anticipation” OR “decision making” OR “perceptual cognitive training”) AND (“intervention” OR “experimental” OR “quasi-experimental” OR “experimental group” OR “control group” OR “training”) AND (“team sport” OR “football” OR “soccer” OR “futsal” OR “handball” OR “volleyball” OR “basketball” OR “hockey” OR “rugby” OR “cricket” OR “water polo” OR “lacrosse” OR “softball” OR “korall” OR “American football”)) and Preprint Citation Index (Exclude − Database) and Article (Document Types)	1200
Scopus	TITLE-ABS+B4-KEY (“decision training” OR “decision making training” OR “anticipation training” OR “video-based training” OR “VBT” OR “video training” OR “perceptual training” OR “video feedback” OR “visual training” OR “vision training” OR “anticipation” OR “decision making” OR “perceptual cognitive training”) AND TITLE-ABS-KEY (“intervention” OR “experimental” OR “quasi-experimental” OR “experimental group” OR “control group” OR “training”) AND TITLE-ABS-KEY (“team sport” OR “football” OR “soccer” OR “futsal” OR “handball” OR “volleyball” OR “basketball” OR “hockey” OR “rugby” OR “cricket” OR “water polo” OR “lacrosse” OR “softball” OR “korall OR “American football”) AND (LIMIT-TO (DOCTYPE, “ar”)) AND (LIMIT-TO (LANGUAGE, “English”)) AND (LIMIT-TO (PUBSTAGE, “final”))	811
EBSCO (SPORTDiscus + PsychInfo)	SU (“decision training” OR “decision making training” OR “anticipation training” OR “video-based training” OR “VBT” OR “video training” OR “perceptual training” OR “video feedback” OR “visual training” OR “vision training” OR “anticipation” OR “decision making” OR “perceptual cognitive training”) AND SU (“intervention” OR “experimental” OR “quasi-experimental” OR “experimental group” OR “control group” OR “training”) AND SU (“team sport” OR football OR soccer OR futsal OR handball OR volleyball OR basketball OR hockey OR rugby OR cricket OR “water polo” OR lacrosse OR softball OR korall OR “American football”) Filters: Full Article, English, Academic Journal, Article.	138
